# Synergistic antibacterial activity between penicillenols and antibiotics against methicillin-resistant *Staphylococcus aureus*

**DOI:** 10.1098/rsos.172466

**Published:** 2018-05-30

**Authors:** Shuihong Li, Qianqian Mou, Xinya Xu, Shuhua Qi, Polly H. M. Leung

**Affiliations:** 1 Hunan Province Cooperative Innovation Center for Molecular Target New Drug Study, Institute of Pathogenic Biology, Medical College, University of South China, Hengyang 421001, People's Republic of China; 2 Key Laboratory of Tropical Marine Bio-resources and Ecology, South China Sea Institute of Oceanology, Chinese Academy of Sciences, Guangzhou 510301, People's Republic of China; 3 Department of Health Technology and Informatics, Hong Kong Polytechnic University, Hong Kong 999077, People's Republic of China

**Keywords:** penicillenols, synergistic antibacterial activity, methicillin-resistant *Staphylococcus aureus*

## Abstract

Penicillenol A2 (isolated from deep-sea fungus *Penicillium biourgeianum* DFFSCS023) has good antibacterial activity against methicillin-sensitive *Staphylococcus aureus* and in combination with *beta*-lactam antibiotics it could significantly decrease methicillin-resistant *Staphylococcus aureus* (MRSA) survival, which provides a novel treatment consideration for MRSA-caused infections.

## 1. Introduction

*Staphylococcus aureus* (*S. aureus*) is a ubiquitous pathogen that commonly colonizes in the nose, respiratory tract and also on the skin. *Staphylococcus aureus* usually survives in a commensal condition with other strains, and it often presents weak pathogenicity [1,2]. However, there were also identified pathogenic *S. aureus* strains, which produce virulence factors that cause a wide variety of diseases from skin infections (abscesses, folliculitis and scalded skin syndrome) to life-threatening disease (meningitis, osteomyelitis, endocarditis, sepsis and toxic shock syndrome) [3,4]. *Beta*-lactam antibiotics are used in the treatment of staphylococcal infections. They can effectively inhibit the growth of *S. aureus* by covalently binding to the transpeptidase domain of penicillin-binding proteins and blocking the formation of the polypeptide cross-links of the bacterial cell wall and subsequently lead to bacterial osmotic lysis [5,6].

At present, antibiotic resistance caused by the widespread use of oral antibiotics has become a serious global health issue [7,8]. The emergence and rapid spread of methicillin-resistant *Staphylococcus aureus* (MRSA) have made the treatment of staphylococcus infections more difficult [9]. MRSA strains were identified to be highly resistant to different categories of *beta*-lactam antibiotics due to the production of *beta*-lactamase, and the expression of penicillin-binding protein 2a (PBP2a, encoded by *mecA*, horizontally acquired from the *SCCmec* cassette) made bacteria more resistant [10,11]. Developing *beta*-lactamase inhibitors (clavulanate and sulbactam) and/or PBP2a inhibitors could be a direction to resolve the resistance of MRSA [12–14].

The ocean covers 70% of the world's surface, and 95% of the area of the ocean was reported greater than 1000 m deep [15]. The enormous deep sea acquired abundant biodiversity [16–18]. Marine organisms were always considered as a rich source of structurally unique, bioactive secondary metabolites. It has been reported that over 30 000 compounds were isolated from marine organisms [19,20]. However, because of the difficulty of marine isolation, only hundreds of compounds were extracted from deep-sea organisms in past decades [21]. We found the extract of *Penicillium biourgeianum* DFFSCS023 exhibited antibacterial effect against methicillin-sensitive *Staphylococcus aureus* (MSSA) through our long-term search for new antibacterial agents from deep-sea fungi. The continental world is running out of antibiotics because bacteria become resistant in a short time. Antibiotics derived from continental species could be one factor. We speculated that the deep-sea-derived antibiotics could reduce bacterial resistance and also have effective antibacterial outcome, which will help us a lot for microbicide development. Photochemistry research of *P. biourgeianum* DFFSCS023 extract concluded that this deep-sea fungus produced 14 compounds (**1–14**) including one novel unknown compound (**1**). Compound **4** exhibited potential antibacterial activity against MSSA in this study. The combination with *beta*-lactam antibiotics could significantly reduce MRSA survival. Herein we report the isolation, elucidation and antibacterial activity of those compounds.

## 2. Material and methods

### 2.1. Fermentation and extraction

The fungus strain DFFSCS024 was isolated from a deep-sea sediment collected at a depth of 2226 m from the South China Sea, Sansha City (17°60' N, 111°48' E), Hainan Province, China. It was identified as *Penicillium biourgeianum* based on the sequence (Genbank accession no. JX156370) of the internal transcribed spacers region of rDNA. Spores were inoculated into 1000 ml Erlenmeyer flasks containing 300 ml of liquid medium (glucose 1%, maltose 2%, monosodium glutamate 1%, KH_2_PO_4_ 0.05%, MgSO_4_ · 7H_2_O 0.003%, corn steep liquor 0.05%, yeast extract 0.3%, dissolved in seawater, pH 6.5). After 35 days of cultivation at 28°C, the broth cultures (a total of 24 l used) were filtered through cheesecloth. Sterilized XAD-16 resin (20 g l^−1^) was added into the previous liquor, and shaken at 200 r.p.m. for 30 min to absorb the organic products. The resin was then washed with distilled water to remove medium residues and eluted with methanol. The methanol solvent was removed under vacuum and subsequently produced a brown residue (approx. 21 g). The mycelium portion was smashed and extracted twice with 80% acetone. The acetone soluble fraction was dried *in vacuo* to yield 20 g of residue. The residues of liquor and mycelium extracts were combined together based on thin-layer chromatography instruction.

### 2.2. Isolation and purification

The combined extract (approx. 40 g) was subjected to silica gel column (900 g), and eluted with CHCl_3_/MeOH (100:0 − 80:20, v/v) to generate 10 fractions (Fr 1–Fr 10). Fr 1 (1.2 g) was isolated with Sephadex LH-20 and purified with HPLC (MeOH/H_2_O, 70:30), and compounds **9** (t_R_37.5 min, 4.8 mg) and **13** (t_R_79.0 min, 4.1 mg) were obtained. Fr 4 (2.5 g) was subjected to an ODS column, and seven sub-fractions (Fr 4.1–Fr 4.7) were obtained. Fr 4.1 was isolated with HPLC (MeOH/H_2_O, 53:47) at the flow rate of 3 ml min^−1^ and compounds **14** (t_R_36.2 min, 56 mg) and **11** (t_R_39.1 min, 3.3 mg) were extracted. Fr 4.3 was purified with HPLC (CH_3_CN/H_2_O, 55:45), and **3** (t_R_77.5 min, 13 mg) and **4** (t_R_81.5 min, 39 mg) were obtained. Fr 4.4 was subjected to HPLC (MeOH/H_2_O, 65 : 35) to obtain compound **10** (t_R_24.3 min, 7.9 mg). Fr 4.6 was also isolated with HPLC (CH_3_CN/H_2_O, 75 : 25) to extract **5** (t_R_43.3 min, 4.1 mg), **8** (t_R_46.7 min, 3.5 mg) and **6** (t_R_53.2 min, 26.8 mg). Fr 4.7 was subjected to Sephadex LH-20 to obtain **1** (7 mg). Fr 6 (0.2 g) was subjected to an ODS column and generated three sub-fractions (Fr 6.1–Fr 6.3). Fr 6.2 was purified with HPLC (MeOH/H_2_O, 65 : 35) to yield compound **12** (t_R_14.2 min, 6.1 mg). Fr 6.3 was isolated with Sephadex LH-20 and purified with HPLC (MeOH/H_2_O, 75 : 25) to extract compound **7** (t_R_23.1 min, 6.8 mg). Fr 5 (0.5 g) was subjected to an ODS column and purified with HPLC (MeOH/H_2_O, 30 : 70) to isolate compound **2** (t_R_12.6 min, 2 mg).

### 2.3. Preparation of the test solutions

The powder of each compound (**4**, **5**, **6**, **8** and **9**) was dissolved in a small volume of dimethylsulfoxide (DMSO) to a final concentration of 8 mg ml^−1^ stock solution, and they were sterilized through a 0.22 μm pore membrane filter. The sterilized stock solutions of each compound were subsequently diluted with TSB buffer to different concentrations for following experimentations. To avoid physiological toxicity, DMSO concentrations were strictly below 0.5% (v/v) [22].

### 2.4. Strains and growth condition

Standard ATCC strain of MSSA ATCC 25923 was used in this study, and MRSA strain was isolated from a patient in Queen Mary Hospital as previously described [23]. Bacteria were grown on Tryptone Soya Agar (TSA; Oxoid, UK), incubated at 37°C overnight. Tryptone Soya Broth (TSB; Oxoid, UK) was used for bacterial broth culture, incubated at 37°C with agitation at 200 r.p.m.

### 2.5. *In vitro* time-kill curve assay

The *in vitro* antimicrobial activities of compounds **4**–**6**, **8–9** against MSSA and **4–6** against MRSA were determined according to Clinical and Laboratory Standards Institute (CLSI, 2004) guidelines. Briefly, the overnight cultures of MSSA and MRSA strains were both diluted to a final concentration of approximately 10^5^ bacteria per ml (CFU ml^−1^), treated with 40 µg ml^−1^ of each of the above compounds and incubated at 37°C for 24 h with 200 r.p.m. shaking. The viable colony counts were determined by the standard plate count method at different time points (0 h, 8 h, 16 h and 24 h).

### 2.6. Combination of antibacterial effect

The overnight cultures of MRSA were diluted to a final concentration of approximately 10^5^ CFU ml^−1^. The diluted MRSA culture was supplied with compound **4** (80 µg ml^−1^), compound **4** (80 µg ml^−1^) and penicillin (20 U ml^−1^), compound **4** (80 µg ml^−1^) and cefotaxime (15 U ml^−1^), compound **4** (80 µg ml^−1^) and oxacillin (2 U ml^−1^), penicillin (20 U ml^−1^), cefotaxime (15 U ml^−1^) and oxacillin (2 U ml^−1^). After incubation for 24 h at 37°C with shaking at 200 r.p.m., the viable bacterial counts of the above different treated cultures were performed. The synergistic antibacterial effects of compounds **5** and **6** were determined as **4**.

### 2.7. Bauer–Kirby disc diffusion method

Antimicrobial susceptibility discs (Oxoid, UK) containing a standard amount of antibiotics were used as positive control, and a negative control with no antibiotics was also set up. To evaluate the antibacterial activities of compounds **4**, **5**, **6**, **8** and **9**, 5 µl or 10 µl of 8 mg ml^−1^ stock solution of each compound were added onto the blank disc (without any chemicals or antibiotics), and dried at 37°C to generate the compound-containing (40 or 80 µg) discs. Similarly, the discs containing a combination of each compound and antibiotics were prepared by adding 5 or 10 µl of the corresponding 8 mg ml^−1^ stock solution onto each previously prepared antibiotics-carrying discs, and dried at 37°C. Single colonies of MSSA and MRSA grown on the TSA plate were, respectively, inoculated into 10 ml of fresh TSB medium, and incubated overnight at 37°C with shaking at 200 r.p.m. A 100 µl aliquot of the overnight bacterial culture suspension (adjusted to a 0.5 McFarland standard) was uniformly spread onto the TSA plates, and the above-prepared discs were each placed on the agar surface. Plates were incubated at 37°C overnight for further antibiotic resistance measurement, and the diameter of inhibition zones was measured through the BIOMIC V3 Microbiology System.

### 2.8. Statistical analysis

All experiments were performed three times. The average values were generated and presented in figures, and the error bars (means ± s.d.) were inserted. *T*-tests (ANOVA) were used to compare the data between groups, and *p* < 0.05 was considered as significant; *p* < 0.01 indicated a very significant difference between groups; *p* > 0.05 was considered as no significance.

## 3. Results and discussion

From our ongoing research aimed at identifying marine natural products, we found one new compound acetic (10*E*,12*E*)-9-oxooctadeca-10,12-dienoic anhydride (**1**) and 13 known compounds: (11*R*)-1-(3-indolyl)-2,3-dihydroxypropan-1-one (**2**), penicillenol A1 (**3**), penicillenol A2 (**4**), penicillenol B1 (**5**), penicillenol B2 (**6**), penicillenol C2 (**7**), demethyllincisterol A2 (**8**), janthinone (**9**), 3,8-dihydroxy-6-methyl-9-oxo-9H-xanthene-1-carboxylic acid methyl ester (**10**), coniochaetone B (**11**), citreorosein (**12**), 6-methylquirizarin (**13**) and decarboxydihydrocitrinone (**14**). The above 14 compounds were all isolated from deep-sea fungus *Penicillium biourgeianum* DFFSCS023. Their chemical structures were elucidated by spectroscopic data.

**Figure 1.**
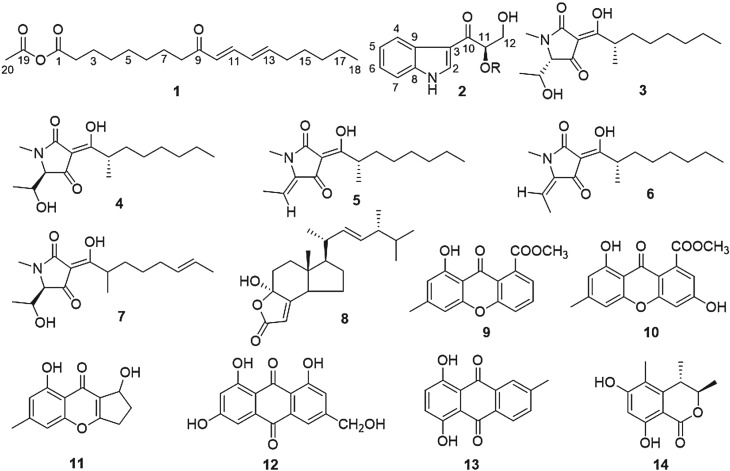
Structures of compounds **1–14**.

Compound **1** was extracted in the form of colourless oil. The HR-ESI-MS analysis (*m/z* 337.2092 [M + H]^+^) uncovered its molecular formula: C_18_H_32_O_4_. The ^1^H NMR spectrum (table 1) showed the presence of four olefinic protons: *δ* 6.07 (1H, d, *J* = 15.5 Hz, H-10), 6.16 (1H, dd, *J* = 10.0, 15.5 Hz, H-12), 6.19 (1H, td, *J* = 6.0, 15.2 Hz, H-13) and 7.12 (1H, dd, *J* = 9.8, 15.5 Hz, H-11); one methyl: 2.03 (3H, s, H-20); and three methylenes: *δ* 2.17 (2H, td, *J* = 6.4, 13.4 Hz, H-14), 2.33 (2H, t, *J* = 7.5 Hz, H-2), 2.53 (2H, t, *J* = 7.4 Hz, H-8), which could occur adjacent to carbonyls or olefinic carbons. The ^1^H NMR spectrum signals at *δ* 0.89 (3H, t, *J* = 6.9 Hz, H-18), 1.20--1.40 (10H, overlapped, H-4, 5, 6, 16, 17), 1.43 (2H, m, H-15) and 1.61 (4H, m, H-3, 7) indicated the presence of a straight carbon chain. The ^13^C NMR data (table 1) of compound **1** were similar to those of the compound 9-oxooctadeca-10,12-dienoic acid except for the presence of a methyl signal at *δ* 22.66 (CH_3_, C-20) and a carboxyl signal at *δ* 173.58 (C, C-19) [24,25]. The configurations of double bonds were determined (10*E*, 12*E*) by coupling constants of H-10 with H-11, H-12 and H-13, respectively. The HMBC correlations from H-20 to C-19 predicted that an acetyl residue could be linked to (*E,E*)-9-oxooctadeca-10,12-dienoic acid at C-1 (figure 1).

**Table 1.** NMR data of compound **1** in CDCl_3_.

	δ_H_ (*J* in Hz)	δ_C_
1		178.11 C
2	2.33 (t, 7.5)	33.85 CH_2_
3	1.62 (m)	24.67 CH_2_
4–6	1.32 (overlapped)	28.88–29.09 CH_2_ × 3
7	1.62 (m)	24.34 CH_2_
8	2.53 (t, 7.4)	40.42 CH_2_
9		201.22 C
10	6.07 (d, 15.5)	127.82 CH
11	7.12 (dd, 9.8, 15.5)	143.12 CH
12	6.16 (dd, 10.0, 15.2)	128.84 CH
13	6.19 (td, 6.0, 15.2)	145.85 CH
14	2.17 (td, 6.4, 13.4)	33.11 CH_2_
15	1.43 (overlapped)	28.38 CH_2_
16	1.32 (overlapped)	31.37 CH_2_
17	1.32 (overlapped)	22.47 CH_2_
18	0.89 (t, 6.9)	14.00 CH_3_
19		173.58 C
20	2.03 (s)	22.66 CH_3_

**Figure 2.**
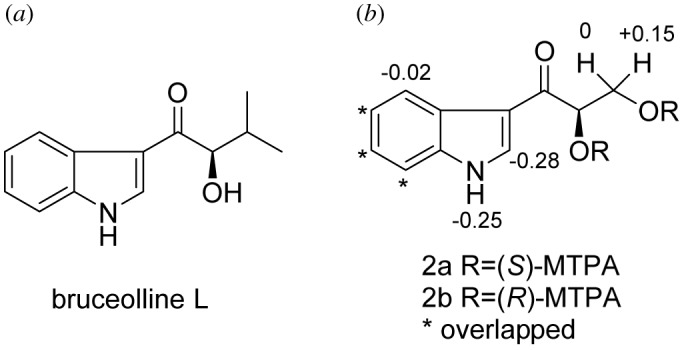
The structures of bruceolline L and Δ*δ* (*δ_S_ −δ*_R_) values for MTPA esters of **2a** and **2b** in CDCl_3_.

Compound **2** was obtained in a colourless amorphous form, and identified as 1-(3-indolyl)-2,3-dihydroxypropan-1-one based on known references [26]. Although its planar structure was reported [27], the whole configuration of 1-(3-indolyl)-2,3-dihydroxypropan-1-one was still unknown. The similar specific rotation data ([*α*]_D_^25^ +58.1 (*c* 0.1, MeOH) for **2**, [*α*]_D_^25^ +19 (*c* 0.05, MeOH) for bruceolline L) and circular dichroism spectrum data (CD (MeOH) *λ*_max_ (log *ϵ*) 240 (0.13), 289 (−0.87), 315 (1.24) nm for **2**; CD (MeOH) *λ*_max_ (log *ϵ*) 247 (−0.07), 281 (−0.43), 313 (0.41) nm for bruceolline L) based on known compound bruceolline L (figure 2) indicated that they had the same configurations at C-11 [28]. Its absolute configuration was confirmed as (11*R*)- by a modified Mosher method Δ*δ*(*δ_S_ −δ*_R_) value distribution pattern [29].

The structures of compounds penicillenol A1 (**3**), penicillenol A2 (**4**), penicillenol B1 (**5**), penicillenol B2 (**6**), penicillenol C2 (**7**) [30], demethyllincisterol A2 (**8**) [31], janthinone (**9**) [32], 3,8-dihydroxy-6-methyl-9-oxo-9H-xanthene-1-carboxylic acid methyl ester (**10**) [33], coniochaetone B (**11**) [34], citreorosein (**12**) [35], 6-methylquirizarin (**13**) [36] and decarboxydihydrocitrinone (**14**) [37] were confirmed through matching spectral data with those of known compounds. Penicillenols **3--7** were first isolated from *Penicillium* sp. GQ-7 [30]. They are *N*-methylated pyrrolidine-2,4-diones (tetramic acid) carrying an α-methyl branched C_8_-fatty acyl residue at the C-3 position. They have shown cytotoxicity [30], and anti-tuberculosis and antimicrobial activity against *Staphylococcus aureus* [38,39].

**Figure 3.**
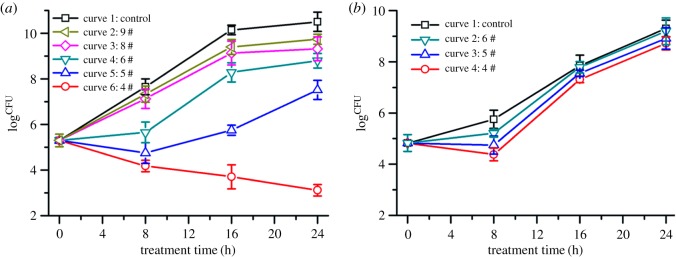
Time-kill curve analysis for *in vitro* evaluation of antibacterial activities of compounds **4**, **5**, **6**, **8** and **9** against MSSA (*a*) and MRSA (*b*). All test concentrations were at 40 µg ml^−1^.

We have chosen 5 of the above compounds (**4**, **5**, **6**, **8** and **9**) to investigate their antibacterial activities on MSSA and MRSA. Other compounds, including the new-found compound (compound **1**) and penicillenol A1 (compound **3**), have almost no antibacterial activities against MSSA and MRSA (these results were not given). The Kirby–Bauer disc diffusion susceptibility test and the *in vitro* time-kill curve assay were performed according to CLSI guidelines. Bauer–Kirby disc diffusion is one of the most commonly used methods of antimicrobial susceptibility assay [40]. The paper discs (6 mm diameter) without antibiotics but containing 40 µg of the compounds were tested, and the zone diameters (ZDs) were read by the BIOMIC V3 Microbiology System. The results (electronic supplementary material, figure S1) revealed that most of the ZDs were too small (ZD ≤ 6 mm) to measure except the ZDs of compound **4** (ZD = 6.75 ± 0.25 mm) and **5** (ZD = 7.5 ± 0.5 mm) against MSSA. Results indicated that these compounds presented weak antibacterial activities that could be caused by less diffusion [41,42]. Furthermore, the *in vitro* time-kill curve assay was necessary to explore the antibacterial activity. As shown in figure 3*a*, all the viable bacteria counts of the supplied compound groups at each time point (8 h, 16 h, 24 h) presented a decline to some degree when compared with negative control without compounds. The antibacterial efficiencies were in the order of compound **4 **> compound **5** > compound **6** > compound **8** > compound **9**.

In addition, compounds **4**, **5** and **6** were selected for the following investigation of the antimicrobial susceptibility to MRSA as described above. There were no significant (*p *> 0.05) differences found between the treating groups and the control groups in MRSA (figure 3*b*). This could be caused by MRSA-produced lactamases, which destroyed the lactam ring of compounds [43].

**Figure 4.**
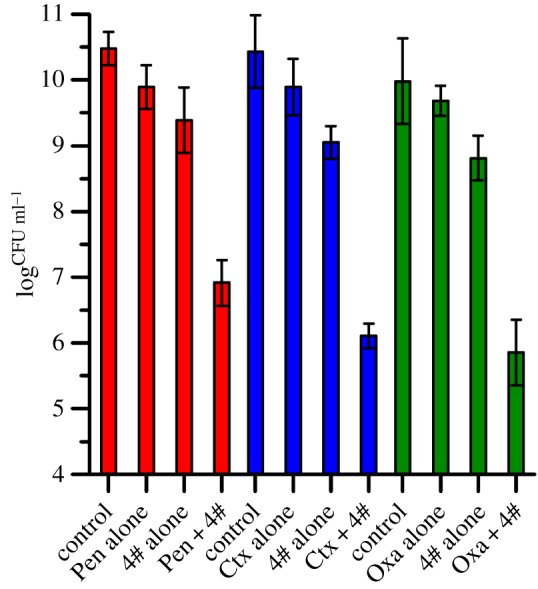
The synergistic antibacterial effect of compound **4** (80 µg ml^−1^) in combination with penicillin (Pen, 20 U ml^−1^), cefotaxime (Ctx, 15 U ml^−1^) and oxacillin (Oxa, 2 U ml^−1^) against MRSA.

Moreover, the synergistic effects of compound **4** associated with penicillin G sodium (Pen), cefotaxime sodium (Ctx) and oxacillin sodium (Oxa) were investigated by plate counting and the Kirby–Bauer disc diffusion method. The synergistic antibacterial effect was defined as the decrease of more than or equal to 2 log^CFU/ml^ between the combination and the single treatment [44,45]. As shown in figure 4, the antibacterial capacity of Pen (10 U ml^−1^), Ctx (15 U ml^−1^) and Oxa (1 U ml^−1^) were all less than 1 log of bacterial CFU per ml when compared with the viable bacteria counts of controls. All the combinations groups between compound **4** and each of the antibiotics (Pen, Ctx and Oxa) presented a decrease of more than or equal to 2 log^CFU/ml^ bacterial count when compared with compound **4** used alone. It can be concluded that the combination of compound **4** with *beta*-lactam antibiotics presented a synergistic effect. As a result, compound **4** could be used in *beta*-lactam antibiotic treatment in MRSA to enhance susceptibility.

**Figure 5.**
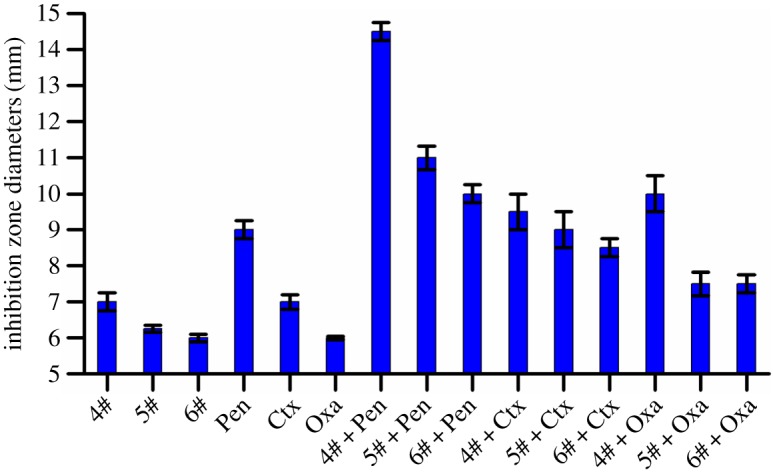
Zone diameter (ZD) histogram of compounds (**4**, **5**, **6**) and antibiotics (Pen, Ctx, Oxa), and the combinations of the two against MRSA. Each disc contained 80 µg of the corresponding compound, antibiotic (Pen, 10 U; Ctx, 30 U; Oxa, 1 U), and the combination groups used the same dosage of each compound with antibiotics (Pen, 10 U; Ctx, 30 U; Oxa, 1 U). ZDs ≤ 6 mm were recorded as 6 mm.

Additionally, the results of the disc diffusion assay revealed that the ZD values of drug combination groups were shown to be larger than each of the ZD values of either penicillenols (compounds **4**, **5** and **6**) or antibiotics (Pen, Ctx and Oxa) used alone, respectively (figure 5). The disc diffusion assay results supported the above outcomes of the synergistic effect between compound **4** and *beta*-lactam antibiotics.

Previous research had demonstrated the antibacterial activities or cytotoxicity of penicillenols and their derivatives [30,39,46], and some penicillenols exhibit antibiofilm activities [47]. However, so far there is no correlative literature about the synergistic effect between penicillenols and antibiotics. So our finding will reveal an avenue for the treatment of MRSA by combining penicillenols and *beta*-lactam antibiotics. Besides, we suspect that the synergistic effect is probably because penicillenols can competitively bind to the *beta*-lactamase or PBP2a of MRSA, or inhibit their biological activities. However, this inference needs further investigation and findings.

## 4. Conclusion

In summary, penicillenols from deep-sea-derived fungus *Penicillium biourgeianum* DFFSCS023 presented antibacterial activity to MSSA, but lower antibacterial capacity against MRSA. A significant synergistic antibacterial effect was found for the combination of penicillenol A2 with either penicillin G sodium, cefotaxime sodium or oxacillin sodium in MRSA treatment. Our discoveries provided a novel choice for clinicians to use penicillenols in combination with *beta*-lactam antibiotics in MRSA infection treatment.

## Supplementary Material

Supplementary Figures

## Data accessibility

The supporting data for this article have been uploaded as part of the electronic supplementary material.
