# Supplementary material for: Synergistic antibacterial activity between penicillenols and antibiotics against methicillin-resistant Staphylococcus aureus
Source: R Soc Open Sci. 2018 May 30;5(5):172466. doi: 10.1098/rsos.172466 (PMC5990757; doi:10.1098/rsos.172466)
Supplement: Supplementary Figures [file rsos172466supp1.doc]

**Synergistic antibacterial activity between penicillenols and antibiotics against methicillin-resistant *Staphylococcus aureus***

Shuihong Li^1^, Qianqian Mou^3^, Xinya Xu^2, *^, Shuhua Qi^2^ and Polly H. M. Leung^3^

1Hunan Province Cooperative Innovation Center for Molecular Target New Drug Study, University of South China, Hengyang 421001, China

2Key Laboratory of Tropical Marine Bio-resources and Ecology, South China Sea Institute of Oceanology, Chinese Academy of Sciences, Guangzhou 510301, China

3Department of Health Technology and Informatics, the Hong Kong Polytechnic University, Hong Kong 999077, China

*Corresponding author. E-mail address: xuxinya@scsio.ac.cn

**Figures contents**

Figure S1. Antimicrobial susceptibility test *(Page 3)*

Figure S2. ^1^H NMR spectrum of compound **1** *(Page 4)*

Figure S3. ^13^C NMR spectrum of compound **1** *(Page 5)*

Figure S4. HSQC spectrum of compound **1** *(Page 6)*

Figure S5. HMBC spectrum of compound **1** *(Page 7)*

Figure S6. HR-ESI-MS spectrum of compound **1** *(Page 8)*

Figure S7. ^1^H NMR spectrum of compound **4** *(Page 9)*

Figure S8. ^1^H NMR spectrum of compound **5** *(Page 10)*

Figure S9. ^1^H NMR spectrum of compound **6** *(Page 11)*

Figure S10. ^1^H NMR spectrum of compound **8** *(Page 12)*

Figure S11. ^1^H NMR spectrum of compound **9**  *(Page 13)*

Figure S12. ^13^C NMR spectrum of compound **5** *(Page 14)*

Figure S13. ^13^C NMR spectrum of compound **6**  *(Page 15)*

Figure S14. Susceptibilities of MSSA and MRSA to antibiotics *(Page 16)*

Figure S15. Identification results of MRSA by Bruker MA LDI Biotyper *(Page 17)*

**
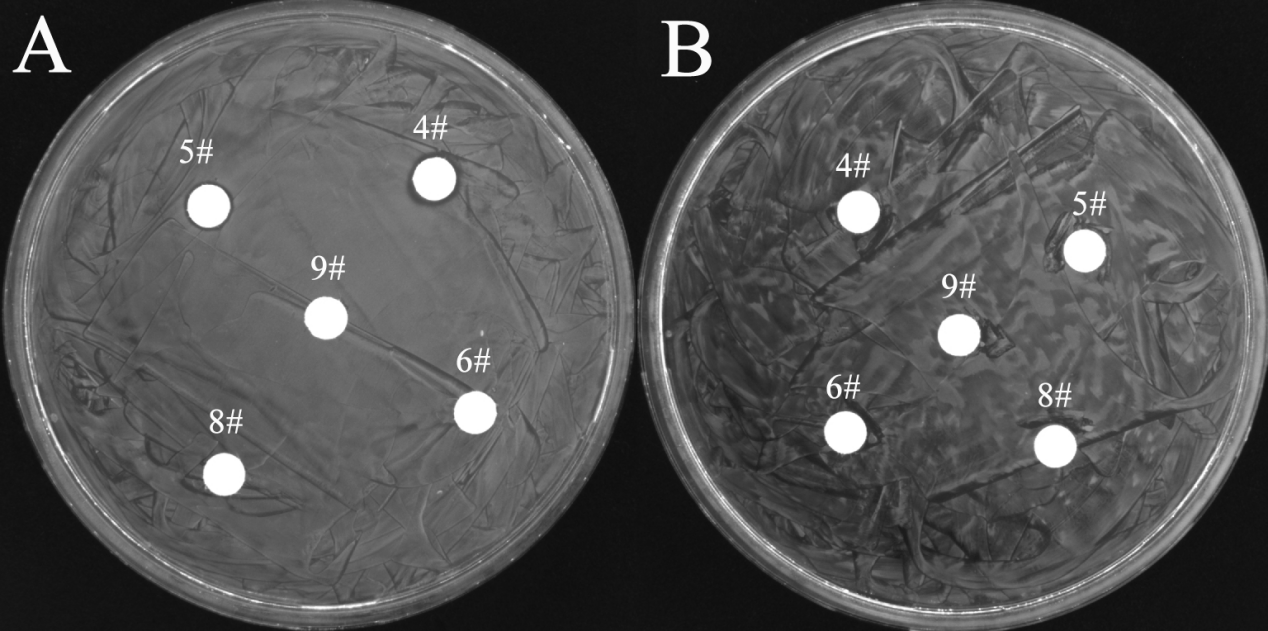
**

**Figure S1.** Antimicrobial susceptibility test of compounds **4**, **5**, **6**, **8** and **9** against MSSA (A) and MRSA (B). All disks contain 40 *μ*g.

**Figure S2.**  ^1^H NMR spectrum of compound **1**.

**Figure S3.**  ^13^C NMR spectrum of compound **1**.


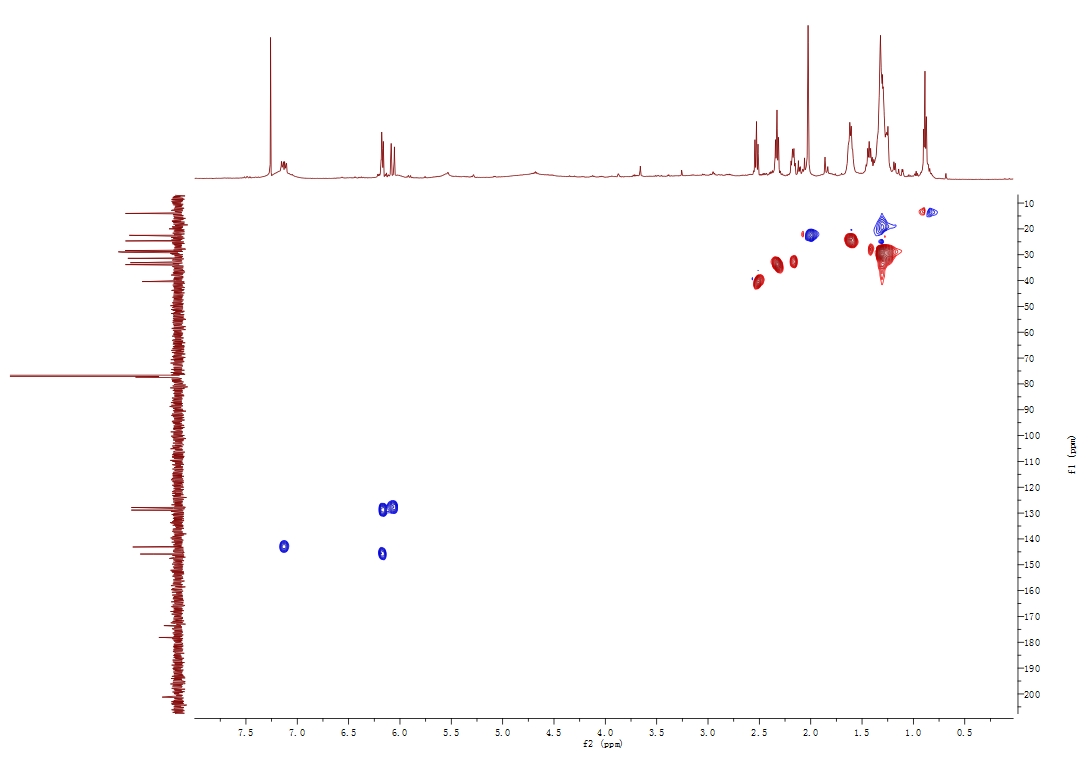


**Figure S4.**  HSQC spectrum of compound **1**.


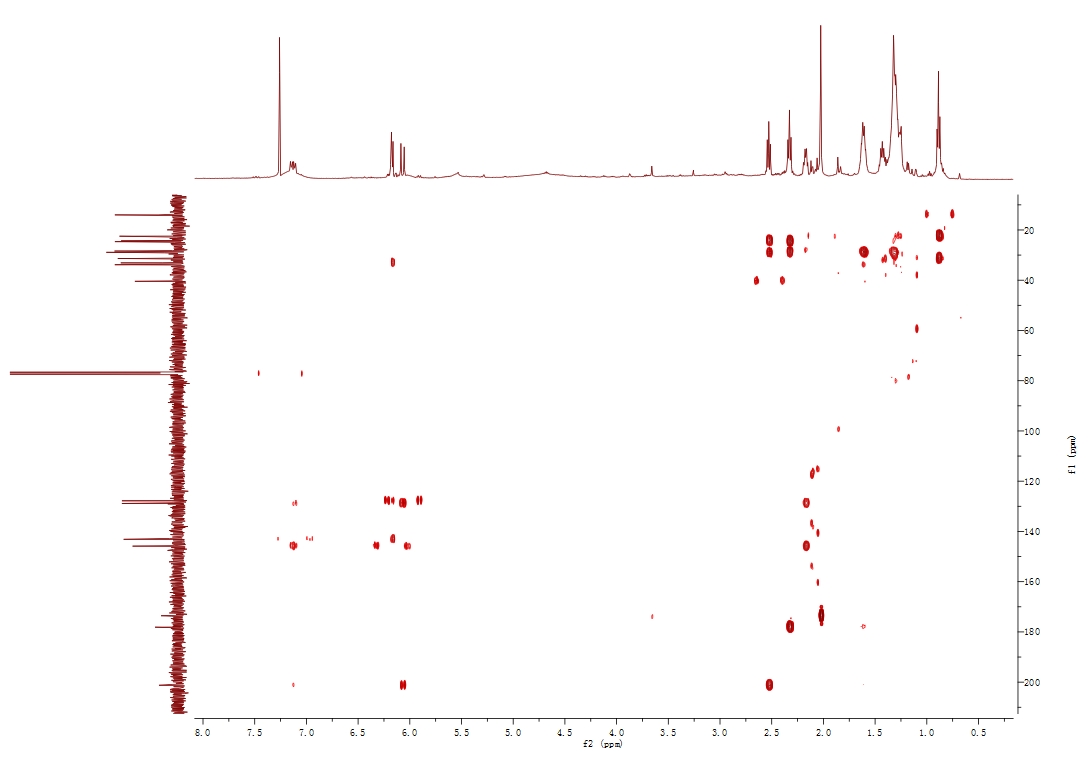


**Figure S5.**  HMBC spectrum of compound **1**.





**Figure S6.**  HR-ESI-MS spectrum of compound **1**.

**Figure S7.** ^1^H NMR spectrum of compound **4**.

**Figure S8.**  ^1^H NMR spectrum of compound **5**.

**Figure S9.** ^1^H NMR spectrum of compound **6**.

**Figure S10.** ^1^H NMR spectrum of compound **8**.

**Figure S11.** ^1^H NMR spectrum of compound **9**.

**Figure S12.** ^13^C NMR spectrum of compound **5**.

**Figure S13.**  ^13^C NMR spectrum of compound **6**.


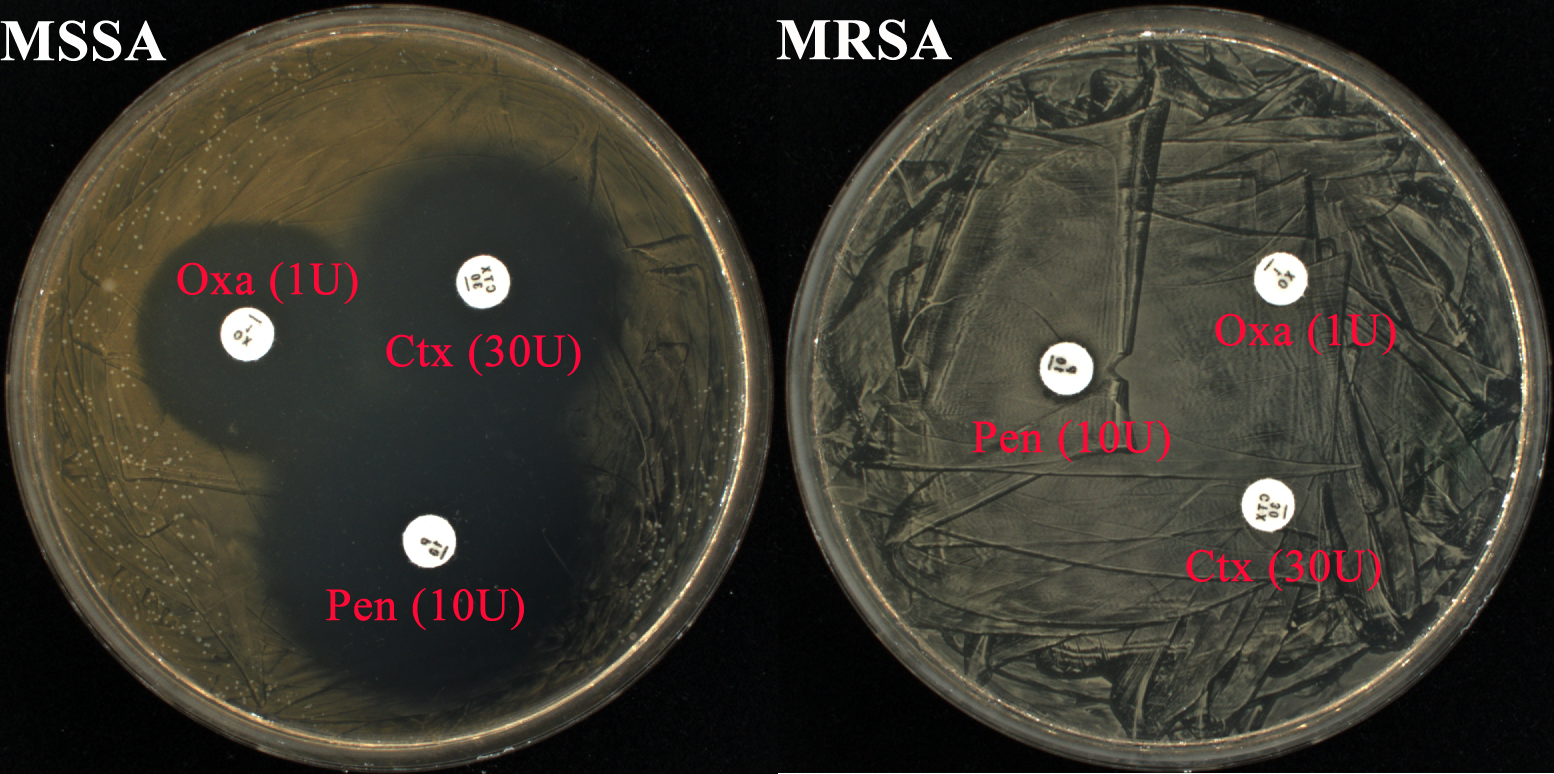


**Figure S14.**  Susceptibilities of MSSA and MRSA to antibiotics (Oxa, 1U; Pen, 10U; Ctx, 30U).

**Sample 1**


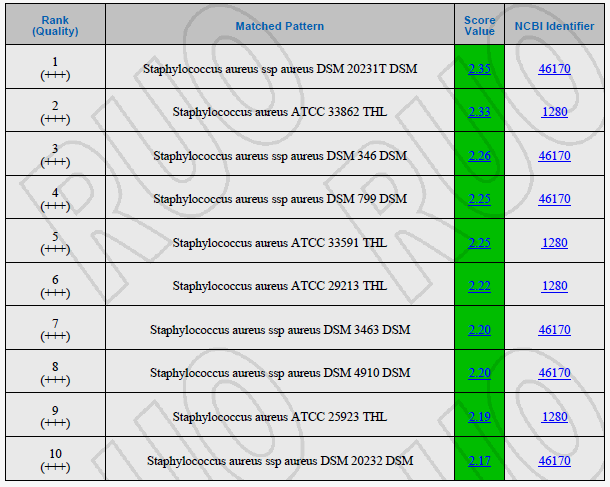


**Sample 2**


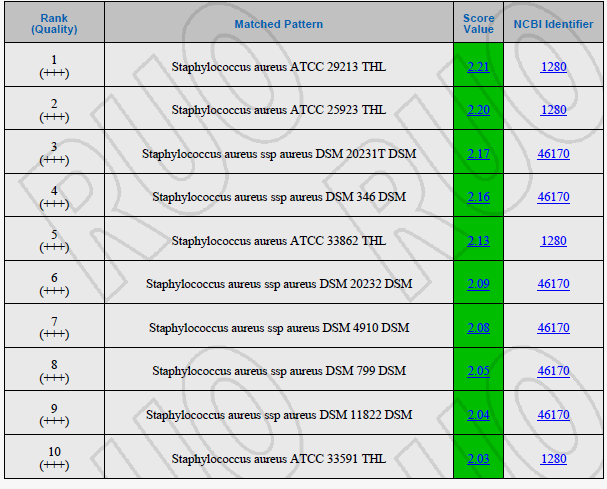


**Figure S15.** Identification results of MRSA by Bruker MA LDI Biotyper. The score values range from “2.0 to 3.0” were marked as “green” and “+++” which means high consistency (the best match is a high-confidence identification). Samples 1 and 2 were in duplicate and both from the MRSA strain.
